# The leaf-air temperature difference reflects the variation in water status and photosynthesis of sorghum under waterlogged conditions

**DOI:** 10.1371/journal.pone.0219209

**Published:** 2019-07-11

**Authors:** Ruidong Zhang, Yufei Zhou, Zhongxiao Yue, Xiaofei Chen, Xiong Cao, Xueying Ai, Bing Jiang, Yifan Xing

**Affiliations:** 1 College of Agronomy, Shenyang Agricultural University, Shenyang, Liaoning, China; 2 Institute of Cash Crops, Shanxi Academy of Agricultural Sciences, Taiyuan, Shanxi, China; Henan Agricultural University, CHINA

## Abstract

Waterlogging stress is one of the most important abiotic stresses limiting sorghum growth and development. Consequently, the responses of sorghum to waterlogging must be monitored and studied. This study investigated changes in the leaf water status, xylem exudation rate, leaf anatomical structure, leaf temperature and photosynthetic performance. Waterlogging-tolerant (Jinuoliang 01, abbreviated JN01) and waterlogging-sensitive (Jinza 31, abbreviated JZ31) sorghum cultivars were planted in pots. The experiment was carried out using a split block design with three replications. Waterlogging stress was imposed at the sorghum five-leaf stage. The leaf free water content (FWC) and relative water content (RWC) decreased under the waterlogged condition. The leaf thickness was thinner under the waterlogged condition, and the main changes occurred in the upper epidermal and mesophyll cells. Gas exchange parameters and the xylem exudation rate were also restrained by waterlogging; however, greater responses of these parameters were observed in JZ31. JZ31 had a higher leaf-air temperature difference (ΔT) than JN01. We found that changes in ΔT were always consistent with changes in the RWC and the gas exchange parameters. ΔT was significantly associated with the leaf RWC, photosynthetic rate (Pn) and transpiration rate (Tr). The results suggest that ΔT may be an indicator reflecting the water status in leaves and can be used to evaluate the tolerance of sorghum to waterlogging.

## Introduction

Waterlogging is becoming an increasingly important abiotic stress that seriously restricts crop growth [1]. Approximately 12% of cropping areas suffer from waterlogging stress every year, and these areas are increasing [2]. Because sorghum has strong resistance to unfavourable conditions, it usually is grown in marginal areas, such as low-lying regions and flooded areas with poor drainage [3]; consequently, sorghum is frequently affected by waterlogging stress due to short-term heavy rainfall. A decline in sorghum growth and therefore a lower yield because of waterlogging have been reported in previous studies [4–5]. To cope with waterlogging stress, the responses of plants to this stress need to be monitored in an accurate and timely manner to potentially mitigate the adverse effects of waterlogging on sorghum production.

The leaf water content is one of most important physiological indicators reflecting the ability to tolerate adversity. The water content is also associated with the root water uptake capacity, leaf transpiration and leaf anatomical structure and is often affected by environmental stresses (such as waterlogging). A marked reduction in the leaf water content of plants under waterlogged conditions was observed in previous studies [6–8]. Setter et al. [9] reported that the water content decreased mainly because the root uptake capacity was restrained due to the lack of oxygen caused by waterlogging. A decreased water content in leaves often affects leaf physiology and metabolism [10]. A leaf water deficit predominantly affects the degree of stomatal opening, resulting in a reduction in leaf transpiration and gas exchange. Reduced photosynthesis and transpiration rates due to a decrease in the leaf water content were observed in maize [11], hibiscus [12], and olives [13]. In addition, the water content is associated with leaf anatomical structures. Yin et al. [14] observed that spongy mesophyll cells were loosely arranged and were able to develop large intercellular spaces in *Dendranthema* leaves under waterlogging stress. The leaf water content plays a vital role in plant growth; however, how this content changes in sorghum leaves under waterlogged conditions is not well known.

Generally, the leaf water content is measured using the oven drying method, electrical impedance and capacitance [15] or a lysimeter [16]. However, these methods are energy intensive, require cumbersome and expensive equipment, and are preferably conducted in the laboratory. Blum et al. [17] monitored the wheat canopy temperature and found that it was significantly correlated with the leaf water content. Changes in leaf temperature can be induced by water loss due to transpiration. Therefore, changes in the leaf water content and transpiration can be indirectly reflected by changes in leaf temperature. With the development of infrared thermography, researchers can accurately and rapidly monitor leaf temperatures using thermal infrared imaging. Wang et al. [18] found that a decrease in the sorghum leaf water content was reflected by thermal infrared images. Although a certain correlation exists between the leaf water content and temperature, the leaf temperature is not only determined by transpiration but is also correlated with air temperature [19]. The temperature difference between the leaf and air (ΔT) may be a more accurate indicator than leaf temperature alone to evaluate the leaf water status [20]. ΔT has been widely used to monitor the leaf water content and leaf gas exchange. However, little information is available in the literature on the water status of sorghum leaves, ΔT and their relationship, especially under waterlogging stress.

Therefore, the aims of this study were to (1) measure changes in the leaf water status, anatomical structure, gas exchange parameters and leaf temperature under waterlogging, (2) identify the relationship between the leaf-air temperature difference and the leaf water status, and (3) confirm that infrared thermography can be used to monitor and evaluate leaf physiological changes in sorghum under waterlogging stress. Monitoring the sorghum leaf water status in a timely and accurate manner is beneficial for developing strategies to cope with waterlogging stress. We also expect that our study will provide a new method for selecting waterlogging-tolerant genotypes for future breeding.

## Materials and methods

### Experimental materials

Jinuoliang 01 (JN01) and Jinza 31 (JZ31) were used as the materials in this study. Based on our previous study, JN01 exhibits tolerance to waterlogging, whereas JZ31 is sensitive to waterlogging. Both cultivars have similar growth periods and plant heights.

### Experimental design

To explore the leaf water status, anatomical characteristics and temperature changes in sorghum under a waterlogged condition, we used a pot culture experimental site at Shenyang Agriculture University, which is located at 41°49’ N and 123°33’ E. This area belongs to the northern temperate zone and has a monsoon-affected semi-humid continental climate. The mean annual temperature is 8°C, the average precipitation is 716.2 mm, and the rainfall is concentrated in June and August, often in the form of rainstorms. The annual frost-free period is 155 to 180 days [21]. The experiment had a split block design with cultivars as the main plot and the waterlogging treatment as the subplot. Every treatment consisted of 30 pots, with one plant per pot. The sorghum was planted in pots (33 cm diameter and 30 cm height) with three holes at the bottom to allow drainage of excess water. Every pot was filled with 19 kg of soil collected from a nearby farm field. The soil contained 30.02 g·kg^-1^ of soil organic matter, 112.27 mg·kg^-1^ of available nitrogen, 8.84 mg·kg^-1^ of rapidly available phosphorus, and 100.13 mg·kg^-1^ of rapidly available potassium. Sorghum seeds were sown on May 14 and emerged on May 21. The plants were watered regularly to maintain optimum soil moisture until the five-leaf stage. The waterlogging treatment was imposed on June 21. According to the experimental design, half of the sorghum pots were waterlogged. These pots were placed in another pot with no holes at the bottom. Then, water was applied, and the water level was maintained at 3 to 5 cm above the soil for two weeks. The control group (CK) were watered as normal. After the waterlogging treatment was completed, the morphology and physiological indicators were measured.

### Measurement of the leaf water status

The leaf water status of the cultivars was measured on July 5 (two weeks after the waterlogging treatment was imposed) at 10:00 am. Ten plants per treatment were used to determine the leaf water status. Leaf discs (8 mm in diameter) were collected from the most recent fully expanded leaf using a sharp leaf punch, and avoiding the major veins. Three hundred leaf discs were collected per treatment (30 discs per leaf). The leaf discs were evenly mixed, and 50 were weighed immediately to record the fresh weight (FW). The leaf discs were placed in distilled water and kept in the dark for 5 h, and then the water on the sample surfaces was dried using absorption paper. The leaf discs were weighed; this weight was recorded as the turgid weight (TW). Next, the leaf discs were placed in different paper bags for each treatment and oven-dried at 105°C for 1 h and then at 80°C until a constant weight was achieved, which was measured as the dry weight (DW). Each treatment was repeated three times. The relative water content (RWC), water saturation deficit (WSD) and total leaf water content (TLC) were calculated according to Singh et al. [22]. The specific leaf weight was calculated according to Zhang et al. [23].

RWC(%)=(FW−DW)/(TW−DW)×100 (1)

WSD(%)=100−RWC(%) (2)

TLC(%)=(FW−DW)/DW×100 (3)

$$Specific\;leaf\;weight\;(g/m^{2})=Total\;dry\;leaf\;weight/Total\;leaf\;area$$ (4)

The free water content (FWC) and bound water content (BWC) were measured according to Zou [24] as follows. A total of 50 leaf discs were placed in a glass bottle fastened with a cap. A glass bottle and cap without leaf discs were weighed and recorded as M1. The leaf discs and bottle were weighed and recorded as M2. Thereafter, 5 ml of a 60% sucrose solution was added to each bottle. The weight of the bottle with the leaf discs and sucrose solution was recorded as M3. The bottles were kept in the dark and gently shaken to ensure that the solution was evenly mixed. After 6 h, the solution concentration was measured using an Abbe Refractometer S2WA-Z (Shanghai, China). The solution concentration was recorded as C, and each measurement was repeated three times.

The FWC and BWC were calculated as follows:

FWC(%)=(M3−M2)×(60%−C)/[(M2−M1)×C]×100 (5)

BWC(%)=TLC(%)−FWC(%) (6)

### Leaf anatomical characteristics

The most recent fully expanded leaf in each treatment was collected, and a 1 cm×1 cm piece was removed from the middle of the leaf (avoiding the main vein) for determination of the anatomical characteristics. The leaf pieces were fixed in a formalin-acetic-alcohol (FAA) solution containing 38% formaldehyde, glacial acetic acid, and 50% alcohol (1:1:18, v/v) for 24 h at 4°C to retain the natural leaf structure according to Du et al. [25]. Fixed leaves were embedded in paraffin, subjected to an ascending dehydration series and then cut into 10-μm slices using an ultra-thin semiautomatic microtome (Ultracut-UC7, Leica, Germany). The slices were fixed onto a glass slide, stained with 1% safranin-O water solution and 0.5% Fast Green solution (dissolved in 95% alcohol) and then observed and photographed under a microscope (ZEISS Axio Scope A1, Germany). The leaf thickness and the upper and lower epidermis cells, vascular bundle (Kranz) area, and xylem vessel number and area were determined and analysed with the ZEN Blue Lite software (n = 25).

### Xylem exudation rates

The xylem exudation rate was measured at 7:00 pm 2 weeks after the waterlogging treatment was imposed. First, absorbent cotton was dried and weighed. Then, the sorghum stem was cut 10 cm above the ground using a sharp knife. Subsequently, the weighed dry cotton was placed on the cut surface. Stem exudates were collected using the absorbent cotton, and the cotton with the exudates was weighed the next morning. To prevent water evaporation, the cotton was covered with a plastic bag. The xylem exudation rate was calculated following Mahmud et al. [26]:

Xylemexudationrate(g/h)=(Weightofcotton+exudates)−(Weightofcotton)/Time (7)

### Gas exchange parameters

The gas exchange parameters were measured using the LI-6400 (LI-COR Biosciences Inc., Lincoln, NE, USA) for the most recent fully expanded leaf in every treatment. The light intensity was set to 1600 μmol (photons)·m^-2^·s^-1^, and the measured gas exchange parameters included the net photosynthetic rate (Pn), stomatal conductance (Gs), intercellular CO_2_ concentration (Ci), and transpiration rate (Tr). The water use efficiency (WUE) was calculated as the ratio of Pn to Tr.

### Thermal infrared image acquisition

Thermal infrared (TIR) images were acquired using the Therma CAM SC 3000 (FLIR Systems, USA), which was equipped with a wide-angle telescope and field of view (FOV) of 20°×15° (width×height); the emissivity coefficient was set to 0.95.

After two weeks of waterlogging treatment, 5 pots were chosen from every treatment and CK group for TIR image acquisition. The images were acquired at 9:00~11:30 using an imager placed above the plants at a distance of 1 metre, and the air temperature was recorded (T_air_). Every image contained one plant. The images were saved as infrared digital images, and every point in the image represented temperature information. After infrared image acquisition, the images were sent to a computer and analysed with the FLIR Tools software. The top three leaves were measured to determine the leaf temperature, and the middle part of each leaf was measured as the monitoring point. The average temperature of the three points was calculated as the leaf temperature (T_leaf_). ΔT was calculated using the following formula:

$$\Delta T=T_{\mathrm{leaf}}-T_{\mathrm{air}}$$ (8)

### Morphological indicators

Morphological indicators were measured after the waterlogging treatment. Three plants were chosen randomly for each treatment. Plant height was measured with a ruler, and then the plants were divided into shoots and roots. The roots were washed with flowing water until clean. The root volume was measured using the water displacement method [27]. Finally, the shoots and roots from each plant were placed into separate paper bags and dried in an oven at 105°C for 1 h and then at 80°C until a constant weight was achieved for measurement of the dry mass (DM).

### Statistical analyses

One-way analysis of variance (ANOVA) and regression analysis were conducted using SPSS 18.0 software. The treatment means were compared based on Duncan’s multiple range test. The results were expressed as the means ± standard deviation (SD).

## Results

### Plant height and dry matter accumulation

**Fig 1:**
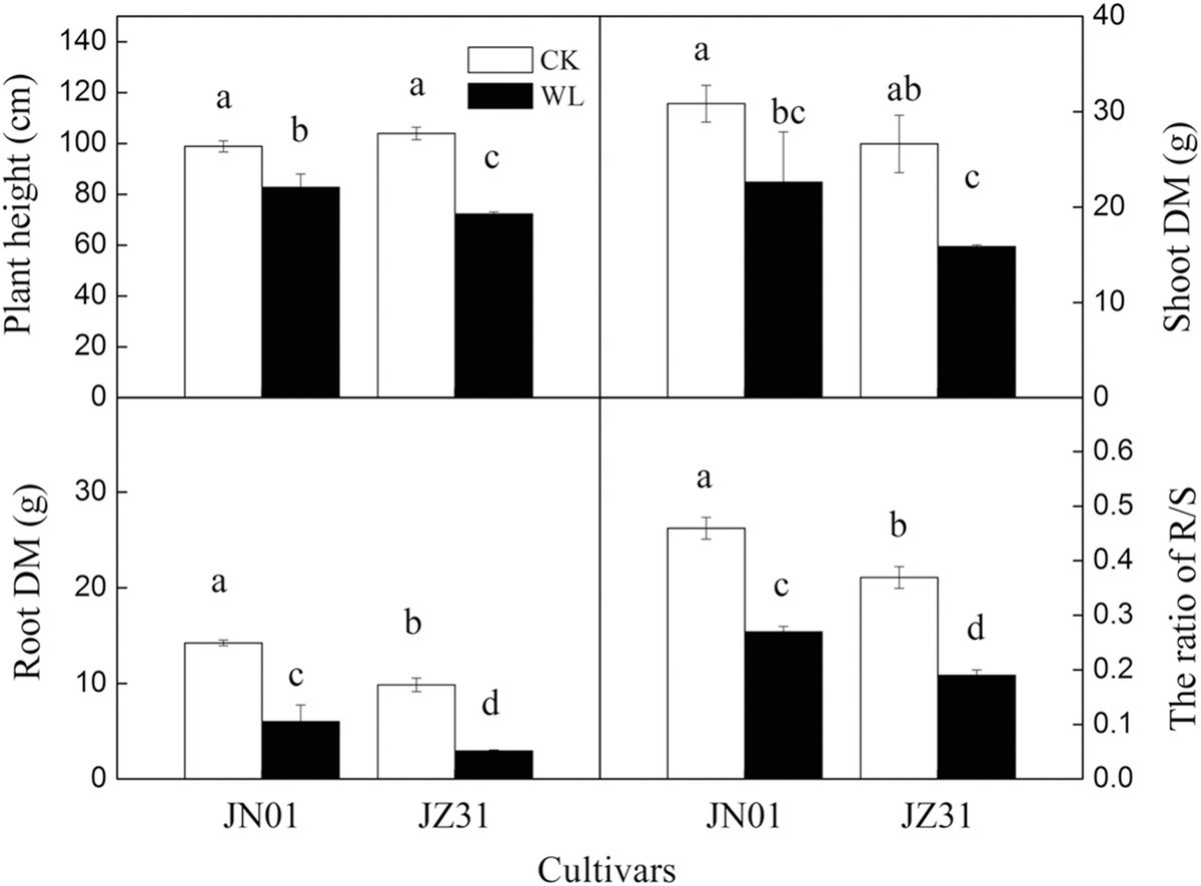
Effect of waterlogging on the plant height and dry mass accumulation of sorghum.

The plant height of both cultivars was significantly restrained by the waterlogging stress; the plant heights of JN01 and JZ31 were decreased by 16.27% and 30.42%, respectively, compared to those of the CK groups. The changes in the shoot and root DM accumulation were similar to those of the plant height; the shoot and root DM declined by 26.64% and 57.87% compared to those of the CK group in JN01 and by 40.41% and 70.10% in JZ31, respectively. Waterlogging stress affected the sorghum roots more severely than the shoots. Therefore, the root to shoot ratios in both cultivars were decreased compared to those of the CK group (by 42.17% in JN01 and 49.98% in JZ31) (Fig 1).

### Leaf water status

Waterlogging resulted in a reduction in the RWC in both cultivars compared with that of the CK groups; the RWC of waterlogged JN01 and JZ31 was reduced by 2.64% and 7.57%, respectively. The reduction in the RWC increased the WSD, ultimately leading to a higher WSD in JZ31 than in JN01 under the waterlogged condition. The FWC was more easily influenced by waterlogging stress than the BWC. Under waterlogging stress, the FWC declined by 11.60% and 19.10% in JN01 and JZ31, respectively, compared to that of the CK groups. The BWC in the two cultivars was not significantly different from that of the CK groups. The FWC/BWC under waterlogging was reduced by 15.15% and 22.8% in JN01 and JZ31, respectively, compared to that of the CK groups (Table 1).

**Table 1:** Effect of waterlogging on the leaf water status in sorghum.

Parameters	JN01		JZ31	
	CK	WL	CK	WL
FWC (%)	31.13±1.88 a	27.52±1.64 ab	30.05±2.88 a	24.31±2.46 b
BWC (%)	47.38±1.45 b	49.22±0.81 ab	48.94±1.39 ab	50.99±0.41 a
FWC/BWC	0.66±0.05 a	0.56±0.03 ab	0.62±0.08 a	0.48±0.05 b
RWC (%)	97.71±0.50 a	95.13±0.38 b	97.94±0.19 a	90.53±1.30 c
WSD (%)	2.29±0.50 c	4.87±0.38 b	2.06±0.19 c	9.47±1.30 a

Notes: CK: control, WL: waterlogging treatment, FWC: free water content, BWC: bound water content, RWC: relative water content, WSD: water saturation deficit. Values are expressed as the mean ± SD of 5 replicates. Values within a row followed by different letters are significantly different (P<0.05) as determined by Duncan’s test.

### Leaf anatomical structures and specific leaf weight

**Fig 2:**
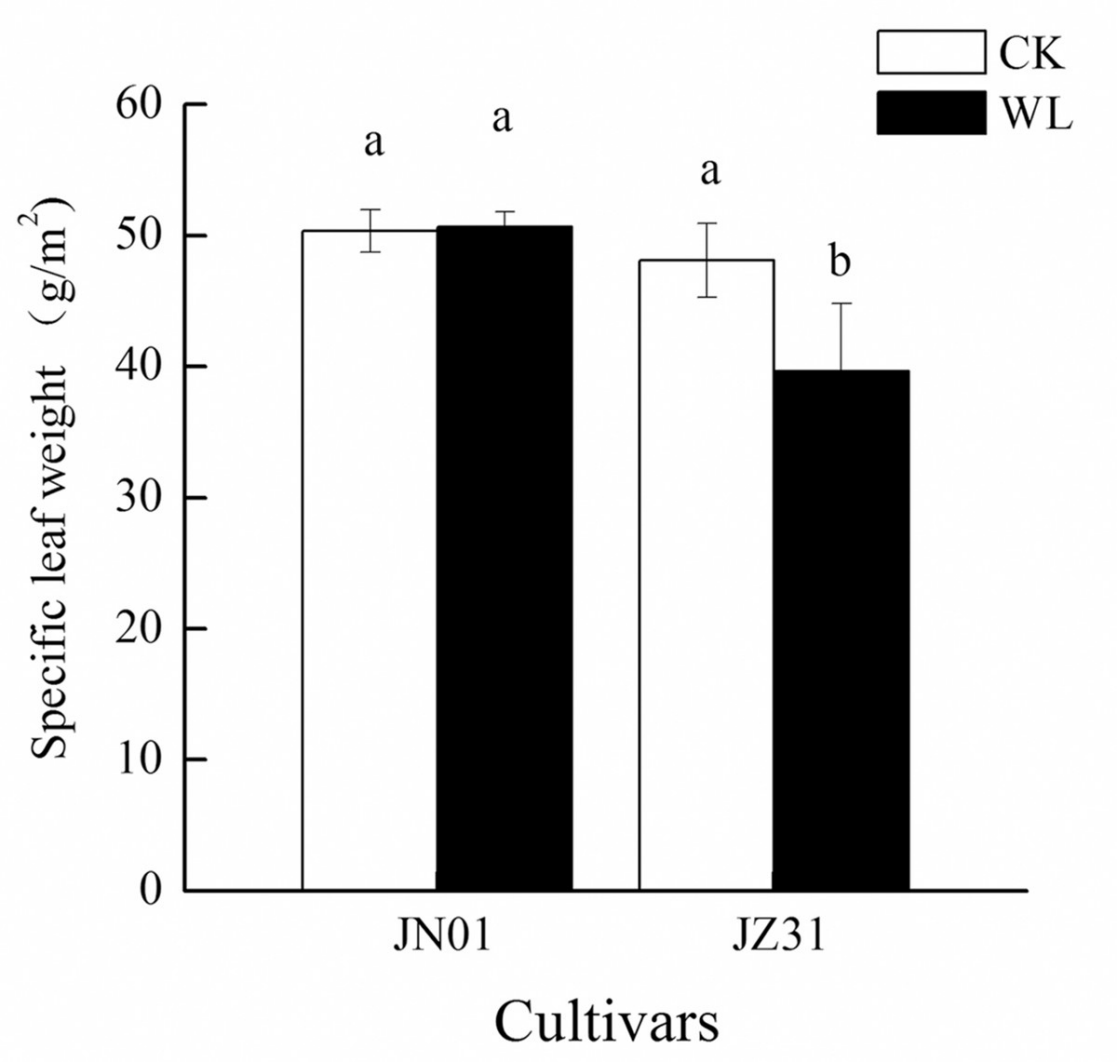
Effects of waterlogging on the specific leaf weight of sorghum.

The leaf anatomical structure changed significantly in both cultivars under waterlogging stress. Microscopic observations showed that the leaf thickness was significantly decreased by 5.04% and 12.84% in JN01 and JZ31, respectively, compared with that of the CK groups. The decrease in leaf thickness was mainly due to changes in the thickness of the upper epidermal and mesophyll cells, and the changes were more obvious in JZ31. The thickness of the upper epidermal and mesophyll cells decreased by 36.98% and 17.19% in JZ31 and by 8.96% and 3.14% in JN01, respectively, compared to those of the CK groups; however, the difference was not significant for JN01. No significant difference in the lower epidermal cells was found between the cultivars. The Kranz area and xylem vessel area did not change significantly in JN01, whereas these parameters decreased by 16.13% and 39.32%, respectively, in JZ31. No significant difference was found in the xylem vessel number (Table 2). The specific leaf weight of JZ31 was decreased by 17.64% compared to that of the CK group. No significant difference in this parameter was found in JN01 between the waterlogging treatment and the CK (Fig 2).

**Table 2:** Leaf anatomical structures of JN01 and JZ31 under the CK and waterlogged conditions.

Parameter	JN01		JZ31	
	CK	WL	CK	WL
Leaf thickness (μm)	188.76±5.23 a	179.24±3.32 b	174.56±7.10 b	152.14±3.48 c
Upper epidermal cells (μm)	33.14±3.36 a	30.17±2.62 a	33.86±4.33 a	21.34±1.66 b
Lower epidermal cells (μm)	22.03±1.55 a	22.17±1.74 a	20.42±1.88 a	20.65±1.85 a
Mesophyll cells (μm)	127.20±3.91 a	123.20±4.90 a	123.65±6.19 a	102.40±6.36 b
Kranz area (μm^2^)	5334.09±368.33 a	5056.41±458.05 a	4891.33±804.82 a	4102.47±573.23 b
Xylem vessel number	3.17±0.75 a	2.67±0.52 a	3.17±1.17 a	2.33±0.82 a
Xylem vessel area (μm^2^)	365.79±35.74 a	362.73±29.25 a	318.45±17.79 b	193.25±48.17 c

Notes: CK: control, WL: waterlogging treatment. Values are expressed as the mean ± SD of 10 replicates. Values within a row followed by different letters are significantly different (P<0.05) as determined by Duncan’s test.

### Root volume and xylem exudation rates

**Fig 3:**
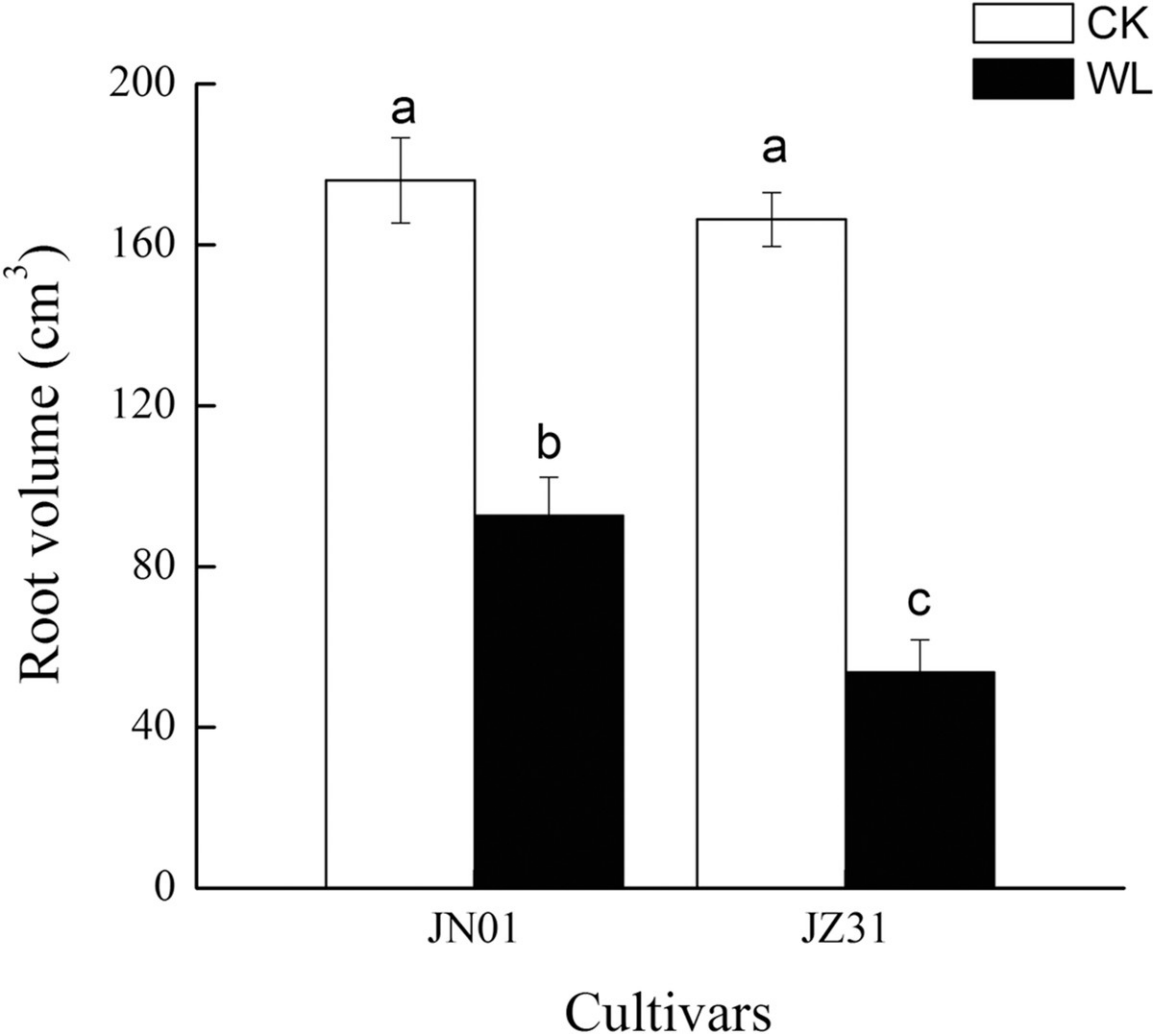
Effect of waterlogging on the sorghum root volume.

**Fig 4:**
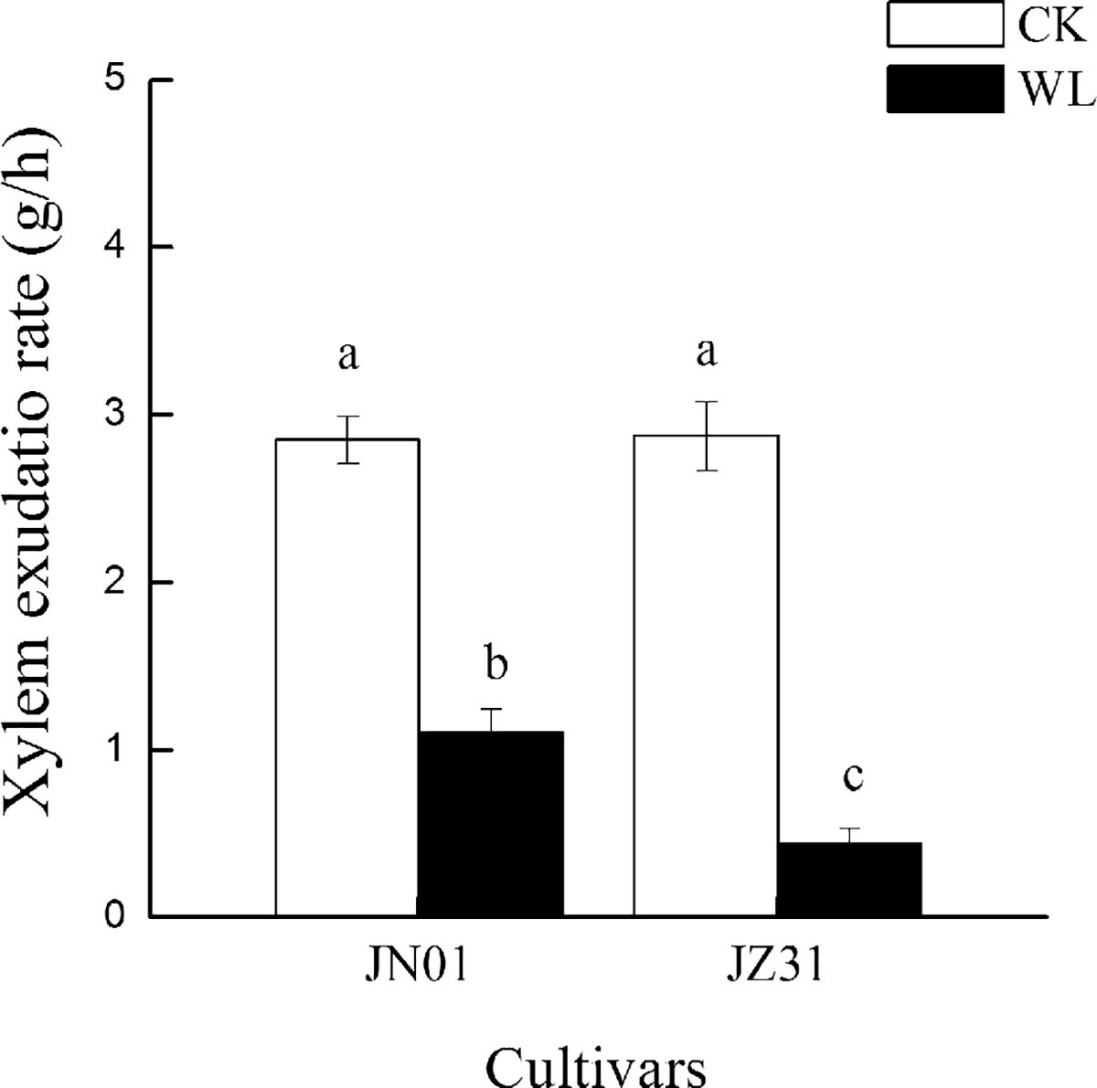
Effect of waterlogging on the xylem exudation rate of sorghum.

Under the CK condition, the root volume did not significantly differ between the two cultivars. With the increase in the waterlogging duration, the root volume in both cultivars became smaller than that of the CK groups. The root volume was reduced by 47.35% in JN01 and by 67.74% in JZ31 compared to that of the CK groups (Fig 3). In addition, waterlogging stress significantly decreased the xylem exudation rate in both cultivars compared with that of the CK groups. Under the waterlogged condition, the xylem exudation rates of JN01 and JZ31 decreased by 61.26% and 84.68%, respectively. JN01 exhibited a higher xylem exudation rate than JZ31 under waterlogging stress (Fig 4).

### Gas exchange parameters

The gas exchange parameters were severely influenced by waterlogging. Pn, Tr, and Gs were decreased by 33.17%, 19.88% and 34.48%, respectively, in JN01 and by 51.92%, 37.80% and 53.33%, respectively, in JZ31 compared to those of the CK groups. The Ci in both cultivars exhibited a slight increase, especially in JZ31. The WUE of JN01 was higher than that of JZ31 under waterlogging stress. The WUE was 16.45% and 22.67% lower in JN01 and JZ31, respectively, compared with that of the CK groups (Table 3).

**Table 3:** Effect of waterlogging on sorghum leaf gas exchange.

Parameters	JN01		JZ31	
	CK	WL	CK	WL
Pn (μmol·m^-2^·s^-1^)	35.91±1.97 a	24.00±1.06 b	34.44±2.29 a	16.56±0.67 c
Tr (mmol·m^-2^·s^-1^)	6.79±0.18 a	5.44±0.23 b	6.56±0.24 a	4.08±0.13 c
Gs (mmol·m^-2^·s^-1^)	0.29±0.01 a	0.19±0.01 b	0.30±0.03 a	0.14±0.01 c
Ci (μmol·m^-2^·s^-1^)	216.05±9.47 c	236.51±5.40 b	226.73±5.14 bc	269.75±10.02 a
WUE	5.29±0.32 a	4.42±0.05 b	5.25±0.39 a	4.06±0.06 c

Notes: CK: control, WL: waterlogging treatment, Pn: net photosynthetic rate, Tr: transpiration rate, Gs: stomatal conductance, Ci: intercellular carbon dioxide concentration, WUE: water use efficiency. Values are expressed as the mean ± SD of 5 replicates. Values within a row followed by different letters are significantly different (P<0.05) as determined by Duncan’s test.

### Temperature difference between leaf and air (ΔT)

**Fig 5:**
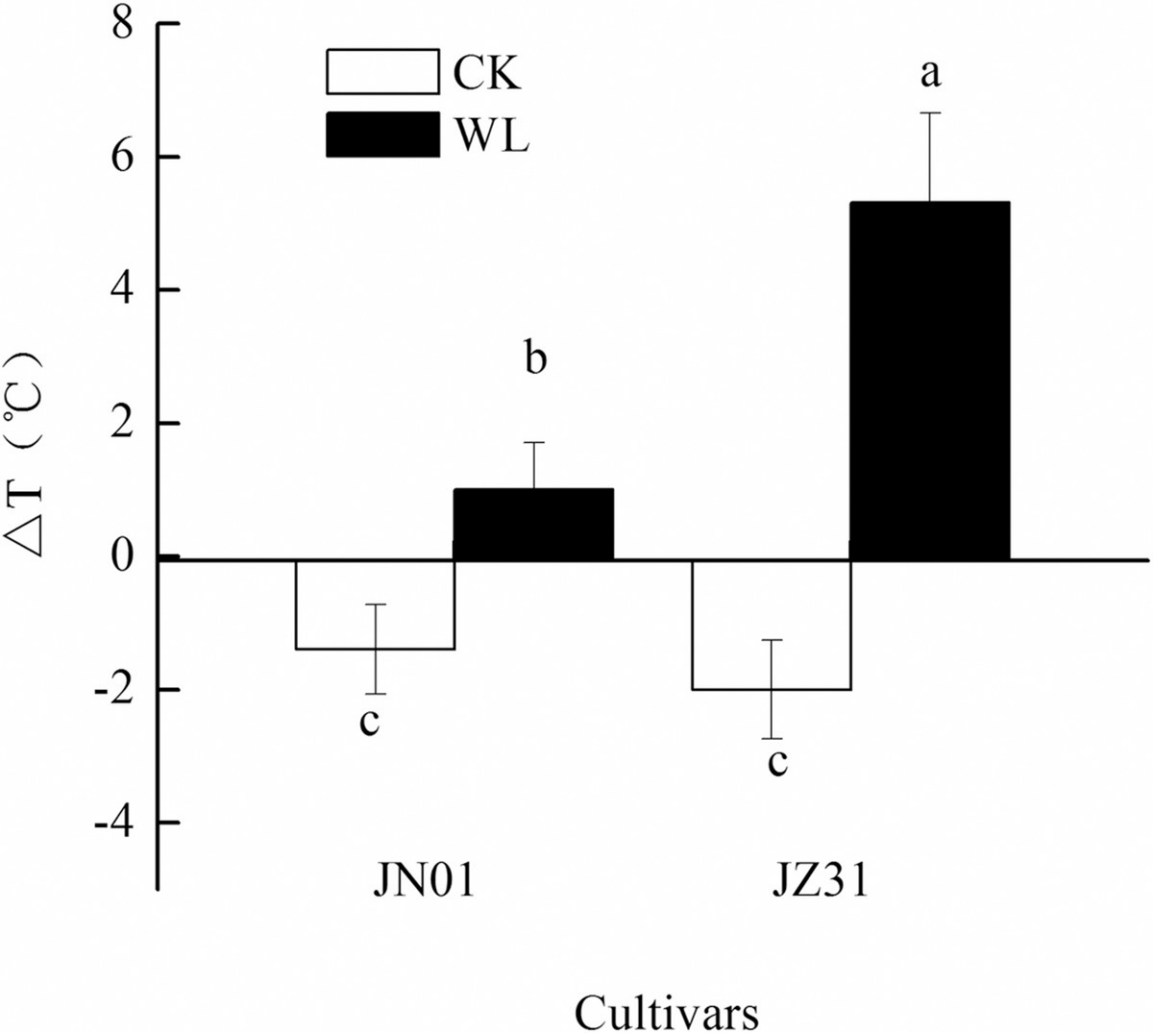
Effect of waterlogging on the ΔT of sorghum.

The effects of waterlogging on the leaf temperature and ΔT are shown in Fig 5. The ΔT of the two cultivars increased under waterlogging stress compared to that of the CK groups. The changes were more obvious in JZ31. The average ΔT value increased by 7.31°C in JZ31 and 2.40°C in JN01 compared to that of the CK groups.

### The relationships between the ΔT and leaf water content and the gas exchange parameters

**Fig 6:**
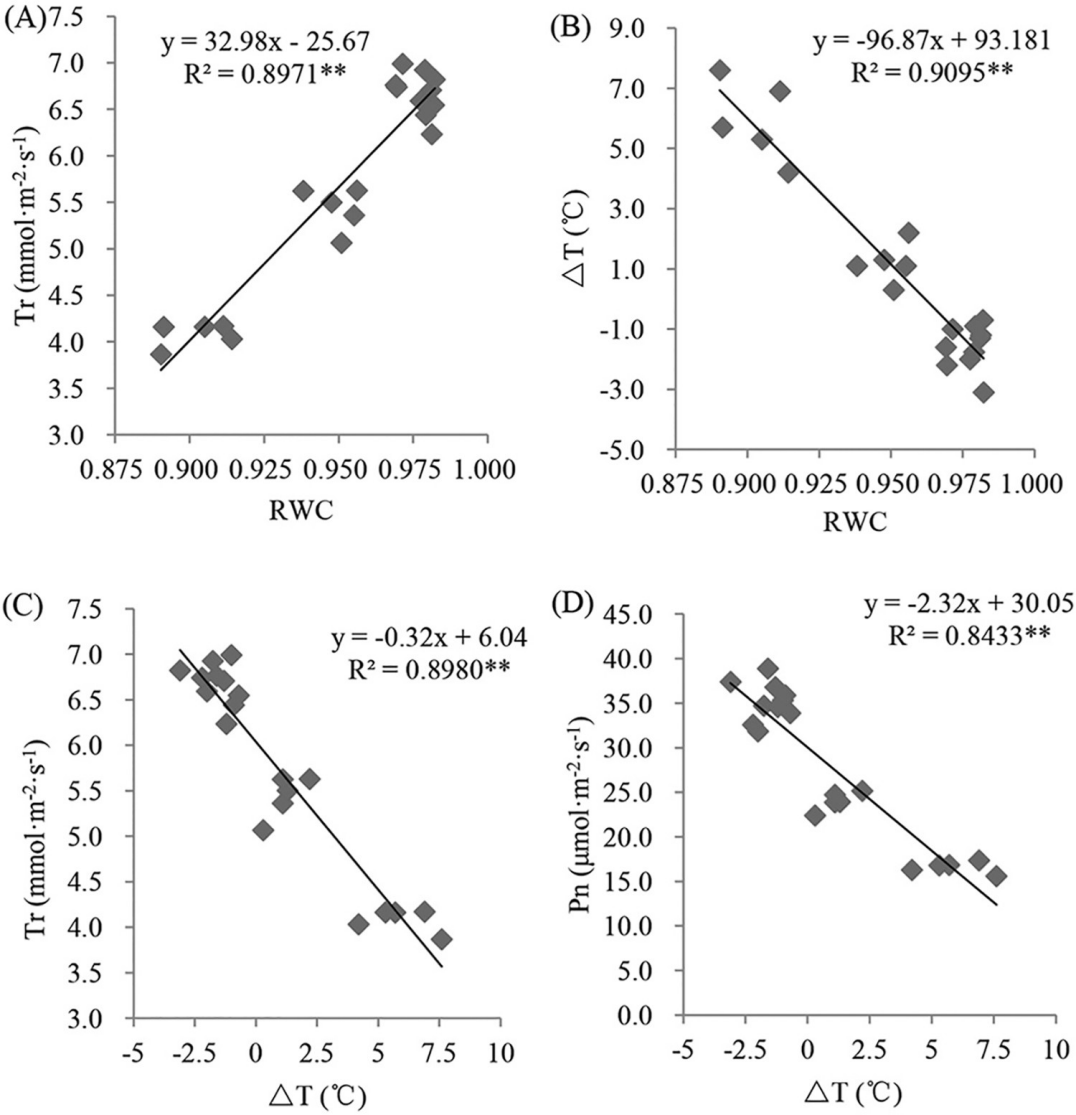
**Relationship between the leaf RWC and Tr (A), leaf RWC and** Δ**T (B),** Δ**T and Tr (C) and** Δ**T and Pn (D) for the pooled samples.** Notes: The data were from the two cultivars under the control and waterlogged conditions, and every treatment included five replicates; **P<0.01; *P<0.05.

A significant positive relationship (R^2^ = 0.8971 P<0.01) was found between the RWC and Tr for the pooled data set (Fig 6A), indicating that the decreasing leaf RWC under waterlogging stress had a negative influence on the leaf Tr. However, a significant negative relationship was found between the RWC and ΔT (Fig 6B), indicating that a lower RWC occurred under a higher ΔT. The Tr decreased as the ΔT increased, and these variables had a significant negative relationship (Fig 6C). Similarly, a negative relationship (R^2^ = 0.9095, P<0.01) was found between Pn and ΔT (Fig 6D), indicating that ΔT reflected changes in Pn and Tr under the waterlogged condition.

## Discussion

When the root growth and function are affected by waterlogging, the shoot growth and function will also be limited. Ren et al. [28] demonstrated that waterlogging substantially restrained the root growth of summer maize and especially decreased the root tip number, root volume, and root length density, thereby restricting photosynthesis and resulting in reduced DM accumulation. Our study found that waterlogging decreased the plant height and DM accumulation in the roots and shoots. The inhibition was more marked in JZ31 than in JN01, indicating that JN01 exhibited higher waterlogging tolerance. Waterlogging usually destroys the crop root system and affects water absorption, resulting in wilting and chlorosis symptoms [29]. In this study, the reduction in the root volume after waterlogging meant that the potential absorption areas for water and nutrients in the roots were decreased, especially in the waterlogging-sensitive cultivar. The xylem exudation rate is an efficient indicator used to reflect the root uptake water capacity [30]. In this experiment, the xylem exudation rate decreased in both of the waterlogged cultivars compared to the CK. This result indicated that sorghum roots were destroyed by waterlogging and that the ability of the root system to absorb water was inhibited. Moreover, we found that the leaf RWC declined in both cultivars under waterlogging stress. This finding was consistent with that reported by Takele et al. [31], who observed that the RWC of cowpea leaves decreased sharply under waterlogging stress. Water in plants is divided into free water and bound water, and the ratio of FWC to BWC is associated with metabolic activity [32]. In this study, we found that waterlogging mainly decreased the FWC but had little influence on the BWC, resulting in a decline in the FWC to BWC ratio. Luan et al. [33] also found that the FWC to BWC ratio in wheat decreased under limited water conditions and that the extent of the decrease was more serious in sensitive cultivars. After waterlogging stress, the FWC to BWC ratio decreased, which might be an adaptation of crops to adversity. In the case of water shortage, the metabolic level of plants declines through a reduction in the FWC to BWC ratio in the leaves, which reduces water consumption to maintain plant survival. In addition, the water in leaves provides turgor pressure for the cells to maintain a proper structure. However, waterlogging decreased the RWC and increased the WSD in the leaves, and the leaf anatomical structure changed accordingly with the WSD. Under the waterlogged condition, the leaf thickness was thinner due to decreased turgor pressure, mainly due to the thinning of the upper epidermal and mesophyll cells, because no significant change was observed in the morphology of the lower epidermal cells. Kulkarni et al. [34] also found that *Ziziphus mauritiana* Lamk. leaves were thinner due to a lack of water. The specific leaf weight, which is expressed as the unit weight per unit leaf area, is a function of the leaf thickness and is positively associated with photosynthesis [35–36]. In this study, the specific leaf weight of JZ31 was decreased due to waterlogging. No significant difference was found in this parameter in JN01 between the waterlogging and CK treatments, implying that the photosynthesis of JZ31 was more influenced.

Leaf stomata are sensitive to the leaf water status, and the stomata are usually closed under waterlogging stress, as shown in previous studies [37–38]. Stomatal closure may decrease gas exchange, especially by affecting leaf transpiration. Transpiration plays an important role in plant growth and development because the impetus of water transport is dependent on the transpiration pull. In addition, transpiration can transport a large amount of heat from the plant leaf surface and plays a vital role in regulating leaf temperature. Although changes in leaf temperature can be used to monitor changes in the leaf water content [39–40], the leaf temperature is also affected by the environment. Temperature differences between leaves and air (ΔT) can reduce the influence of air temperature changes on leaf temperature based on monitoring of the leaf water status [41]. Our study showed that ΔT increased in both cultivars under waterlogging stress, although the change was more obvious in JZ31. We also found that the leaf water content and gas exchange parameters of sorghum changed under waterlogging stress. Regression analysis showed that the RWC and Tr were positively correlated, indicating that the transpiration rate decreased as a result of the lower RWC. We also found that ΔT and Tr were negatively correlated, indicating that the decreased Tr caused the increased ΔT. Moreover, a negative relationship existed between ΔT and Pn. The increase in leaf temperature caused by a water shortage creates unfavourable conditions for plants; the abnormal leaf temperature will disturb physiological metabolism, destroy the chloroplast structure and decrease the Pn [42–44]. Surendar et al. [45] reported that leaf temperature was significantly negatively correlated with the photosynthetic rate. We deduce that a relationship exists between ΔT and the leaf water content and leaf gas exchange parameters; this relationship is a real-time response and is indicated by an extremely significant correlation. Therefore, we believe that ΔT can be used as an index to reflect the water status and photosynthetic physiological characteristics of sorghum leaves after waterlogging. Moreover, because of its convenient, real-time, and non-destructive characteristics, we recommend that ΔT be used as an important means to monitor sorghum waterlogging.

## Conclusion

The results showed that waterlogging severely affected sorghum root function. As a result of changes in the leaf water status, especially decreases in the FWC, the Gs, Tr and Pn declined, resulting in a decreased photosynthetic capability. These indicators decreased more sharply in JZ31 than in JN01. The leaf temperature increased due to the decrease in Tr. ΔT had a certain correlation with the leaf water status and leaf photosynthetic characteristics and could be used as an indicator to evaluate the waterlogging tolerance of sorghum. This study provides important information for selecting waterlogging-tolerant varieties of sorghum or other crops in future breeding programmes.

## Supporting information

S1 Fig Leaf anatomical structures in JN01 and JZ31 grown under the CK and waterlogged conditions. Notes: UC, upper epidermal cells; LC, lower epidermal cells; Kr, Kranz; Ve, vessel; St, stoma.
(TIF)

S2 Fig Thermal infrared images of JN01 and JZ31 leaves grown under the CK and waterlogged conditions. (TIF)

S1 Table The raw data. The table matched to Fig 1.
(XLS)

S2 Table The raw data. The table matched to Table 1.
(XLS)

S3 Table The raw data. The table matched to Table 2.
(XLS)

S4 Table The raw data. The table matched to Fig 2.
(XLS)

S5 Table The raw data. The table matched to Fig 3.
(XLS)

S6 Table The raw data. The table matched to Fig 4.
(XLS)

S7 Table The raw data. The table matched to Table 3.
(XLS)

S8 Table The raw data. The table matched to Fig 5.
(XLS)

S9 Table The raw data. The table matched to Fig 6.
(XLS)
