# Supplementary figures and images for: The leaf-air temperature difference reflects the variation in water status and photosynthesis of sorghum under waterlogged conditions
Source: PLoS One. 2019 Jul 11;14(7):e0219209. doi: 10.1371/journal.pone.0219209 (PMC6624001; doi:10.1371/journal.pone.0219209)

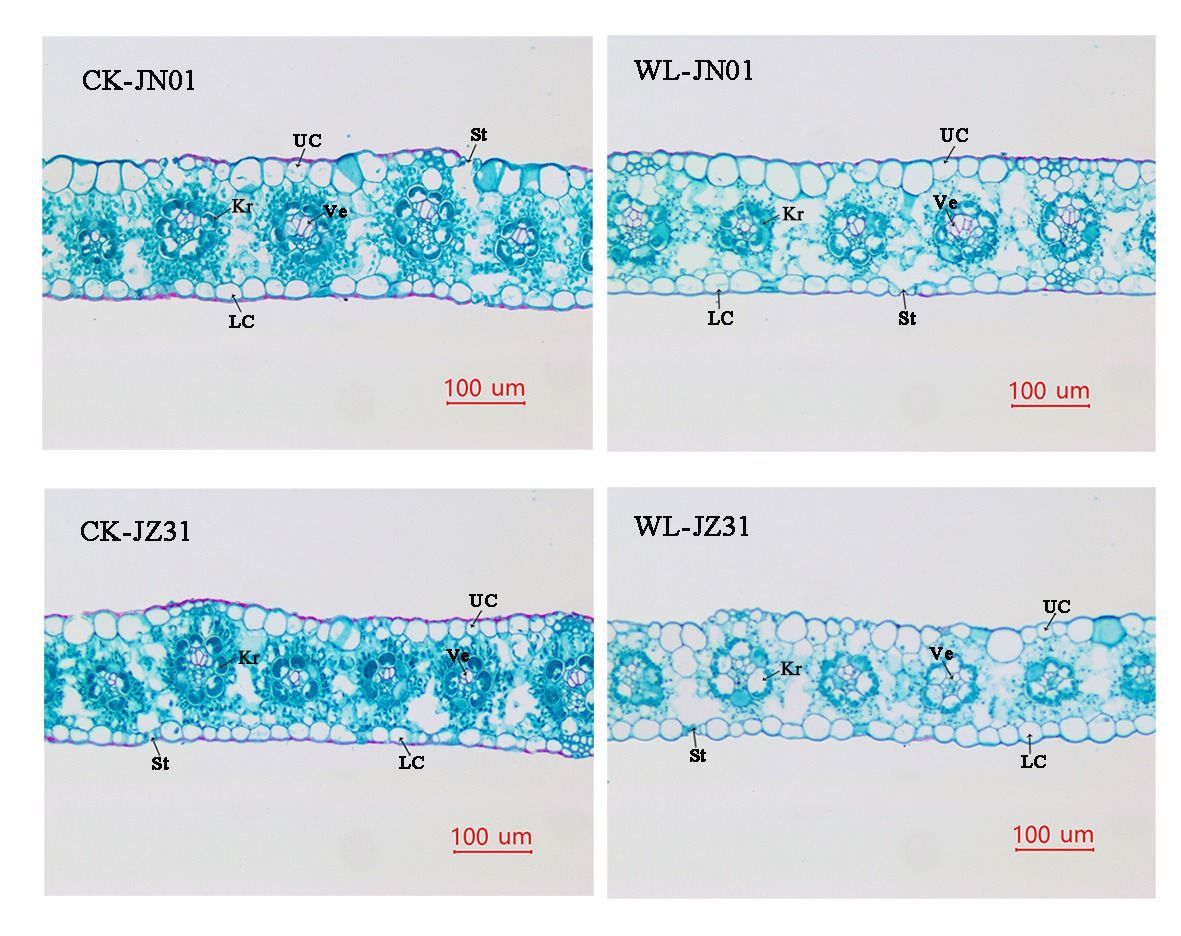

Supplement: S1 Fig — Notes: UC, upper epidermal cells; LC, lower epidermal cells; Kr, Kranz; Ve, vessel; St, stoma. (TIF) [file pone.0219209.s001.tif]

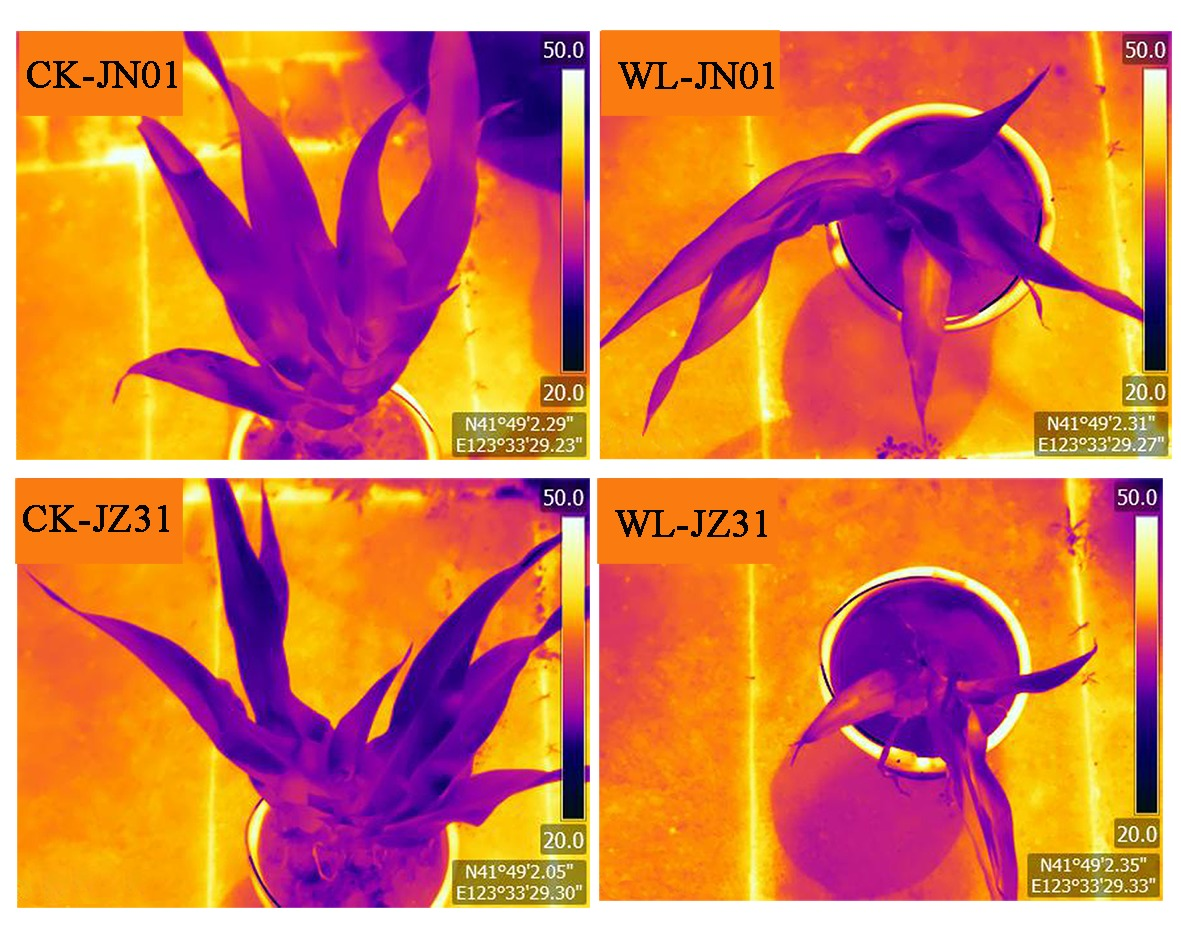

Supplement: S2 Fig — (TIF) [file pone.0219209.s002.tif]
